# Influence of Antihypertensive Treatment on RAAS Peptides in Newly Diagnosed Hypertensive Patients

**DOI:** 10.3390/cells10030534

**Published:** 2021-03-03

**Authors:** Annina S. Vischer, Gabriela M. Kuster, Raphael Twerenbold, Otmar Pfister, Qian Zhou, Andrea Villiger, Marko Poglitsch, Stephan Krähenbühl, Michael Mayr, Stefan Osswald, Manuel Haschke, Thilo Burkard

**Affiliations:** 1 Hypertension Clinic, Medical Outpatient Department and Hypertension Clinic, ESH Hypertension Centre of Excellence, University Hospital Basel, 4031 Basel, Switzerland; michael.mayr@usb.ch (M.M.); thilo.burkard@usb.ch (T.B.); 2 Clinic of Cardiology, University Hospital Basel, 4031 Basel, Switzerland; gabriela.kuster@usb.ch (G.M.K.); raphael.twerenbold@usb.ch (R.T.); otmar.pfister@usb.ch (O.P.); qian.zhou@usb.ch (Q.Z.); stefan.osswald@usb.ch (S.O.); 3 Department of Biomedicine, University Hospital Basel and University of Basel, 4031 Basel, Switzerland; 4 Cardiovascular Research Institute Basel (CRIB), University Hospital Basel, 4031 Basel, Switzerland; 5 Division of Clinical Pharmacology & Toxicology, University Hospital Basel, 4031 Basel, Switzerland; andrea.villiger@bayer.com (A.V.); stephan.kraehenbuehl@usb.ch (S.K.); manuel.haschke@insel.ch (M.H.); 6 Attoquant Diagnostics GmbH, 1110 Vienna, Austria; marko.poglitsch@attoquant.com; 7 Division of Clinical Pharmacology & Toxicology, University Hospital Bern, 3010 Bern, Switzerland

**Keywords:** arterial hypertension, renin–angiotensin–aldosterone system, antihypertensive drug, angiotensin-converting-enzyme inhibitor, angiotensin receptor antagonist, calcium channel blocker, thiazide diuretic

## Abstract

(1) Background: Recently, influences of antihypertensive treatment on the renin–angiotensin–aldosterone system (RAAS) has gained attention, regarding a possible influence on inflammatory and anti-inflammatory pathways. We aimed to study the effects of newly initiated antihypertensive drugs on angiotensin (Ang) II and Ang (1–7) as representers of two counter-regulatory axes. (2) Methods: In this randomized, open-label trial investigating RAAS peptides after the initiation of perindopril, olmesartan, amlodipine, or hydrochlorothiazide, Ang II and Ang (1–7) equilibrium concentrations were measured at 8 a.m. and 12 a.m. at baseline and after four weeks of treatment. Eighty patients were randomized (1:1:1:1 fashion). (3) Results: Between the four substances, we found significant differences regarding the concentrations of Ang II (*p* < 0.0005 for 8 a.m., 12 a.m.) and Ang (1–7) (*p* = 0.019 for 8 a.m., <0.0005 for 12 a.m.) four weeks after treatment start. Ang II was decreased by perindopril (*p* = 0.002), and increased by olmesartan (*p* < 0.0005), amlodipine (*p* = 0.012), and hydrochlorothiazide (*p* = 0.001). Ang (1–7) was increased by perindopril and olmesartan (*p* = 0.008/0.002), but not measurably altered by amlodipine and hydrochlorothiazide (*p* = 0.317/ 0.109). (4) Conclusion: The initiation of all first line antihypertensive treatments causes early and distinct alterations of equilibrium angiotensin levels. Given the additional AT1R blocking action of olmesartan, RAAS peptides shift upon initiation of perindopril and olmesartan appear to work in favor of the anti-inflammatory axis compared to amlodipine and hydrochlorothiazide.

## 1. Introduction

Arterial hypertension (AHT) is the most prevalent modifiable risk factor for premature death worldwide [1]. In context of the ongoing pandemic with severe acute respiratory syndrome coronavirus 2 (SARS-CoV-2), the renin–angiotensin–aldosterone system (RAAS) has gained increasing attention due to their link to inflammatory and anti-inflammatory pathways and since concerns were raised regarding a potential influence of angiotensin-converting-enzyme (ACE)-inhibitors (ACE-I) and angiotensin-receptor blockers (ARB) on the susceptibility to infection with SARS-CoV-2 and on the prognosis of corona virus disease 2019 (COVID-19) [2,3,4]. 

The RAAS mainly consists of two counter-regulatory axes, i.e., the ACE/angiotensin II (Ang II, Ang (1–8))/angiotensin type 1 receptor (AT1R) axis, which mainly promotes vasoconstriction, inflammation, salt and water reabsorption, and the counteractive ACE2/Ang (1–7)/Mas receptor (Mas R) axis, which has a cardioprotective and anti-inflammatory effect [5]. Both axes are connected by the monocarboxypeptidase ACE2, which degrades Ang II into Ang (1–7) and Ang I (1–10) into Ang (1–9) [5]. Besides the well-known cardiovascular effects, both counter-active axes are postulated to play a role in the pathophysiology of acute lung injury and acute respiratory distress syndromes (ARDS), including severe respiratory syndrome (SARS).

Clinicians and hypertensiologists found themselves on the one hand confronted with numerous questions from patients and colleagues, whether or not treatment with ACE-I or ARB should be discontinued or replaced with a putatively safer treatment [4], but on the other hand data about the influence of other first line antihypertensives on the RAAS system, especially Ang II and Ang (1–7) in humans is scarce. Therefore, the aim of this paper was to study the effect of recently initiated antihypertensive drugs on the equilibrium concentrations of Ang II and Ang (1–7) as markers of the pro-inflammatory ACE/Ang II/AT1R axis and the counteractive ACE2/Ang (1–7)/Mas R axis in newly diagnosed hypertensive patients.

## 2. Methods

### 2.1. Study Setting

This is an explorative analysis of a randomized, open-label, parallel-group trial investigating RAAS peptide equilibrium concentrations and non-invasive hemodynamics after initiation of antihypertensive treatment, conducted at the Hypertension Clinic of the Medical Outpatient Department of the University Hospital Basel, Switzerland between April 2015 and March 2017.

### 2.2. Intervention Group

Eighty patients ≥18 years of age diagnosed with primary grade I or II AHT, confirmed by ambulatory blood pressure monitoring (ABPM) requiring antihypertensive drug treatment were consecutively recruited. Baseline ABPM was carried out prior to patient inclusion and was mandatory for the confirmation of AHT and as a baseline measurement (Supplementary Figure S1).

The inclusion and exclusion criteria can be found in Data Supplement 1. Diagnostic work-up was performed according to the current clinical guidelines at the time of inclusion [6]. Patients were randomized in a 1:1:1:1 fashion to either and ACE-I (5 mg of perindopril), an ARB (20 mg of olmesartan), a calcium channel blocker (CCB, 5 mg of amlodipine) or 25 mg of hydrochlorothiazide (HCT).

### 2.3. Normotensive Control Group

Twenty age- and sex-matched healthy and normotensive individuals were included as control group. The recruitment of the control group was carried out in a 4:1 fashion. Normotension was confirmed by ABPM.

### 2.4. Blood Pressure Measurements

A description of the blood pressure (BP) study procedures has been published previously [7]. In brief, ABPM was mandatory for the intervention group prior to inclusion. ABPM was performed and completed during the routine clinical work-up of the patients and performed according to clinical recommendations [6]. ABPM monitors were programmed for measurements every 20 min from 6 a.m. to 10 p.m. and every 30 min from 10 p.m. to 6 a.m. Participants were asked to note their time in bed, which was used to define awake and asleep times.

ABPM values were calculated as a 24 h mean over all values, and additionally, we calculated awake and asleep values. AHT was defined as a 24 h mean systolic BP (sBP) ≥130 mmHg and/or diastolic BP (dBP) ≥ 80 mmHg, awake sBP ≥ 135 mmHg and/or dBP ≥ 85 mmHg, and/or asleep sBP ≥ 120 mmHg and/or dBP ≥ 70 mmHg [6]. Results were scrutinized to detect newly diagnosed AHT.

For the normotensive group, we asked apparently normotensive, age- and sex-matched volunteers to wear an ABPM device to confirm normotension before enrolment.

### 2.5. RAAS Peptide Sampling

Venous blood was collected from each participant into 6.0 mL polypropylene tubes containing heparin at 8 a.m. and 12 a.m. before initiation of the antihypertensive treatment (baseline) and after four weeks of treatment (treatment period; TP). At TP, blood was drawn before drug intake (8 a.m.) and 4 h after observed drug intake (12 a.m.). In the normotensive group, blood was collected at baseline at 8 a.m. and 12 a.m. There was no second phlebotomy.

All samples were immediately centrifuged and stored at −20 °C until analysis. Equilibrium angiotensin concentrations were determined by Attoquant Diagnostics GmbH, Vienna, Austria by a liquid chromatography-tandem mass spectrometry method as described previously [8,9,10]. On study days, patients were fasted (including alcohol consumption) for 10 h before observed drug intake at 8 a.m. A light breakfast was allowed after drug intake. No concomitant drug was allowed.

### 2.6. Trial Registration

The trial was conducted according to the ethical guidelines of the Declaration of Helsinki and the applicable International Conference on Harmonization (ICH) guidelines on good clinical practice. The trial was approved by the local ethics committee and registered on www.ClinicalTrials.gov (accessed on 2 March 2021) (NCT02449811). Anonymized data supporting the findings of this study are available from the corresponding author upon reasonable request.

### 2.7. Statistical Analyses

Continuous data were assessed for normal distribution with box plots. The means of normally distributed data of the four groups were compared using the one-way ANOVA and in case of violated homogeneity of variances, the Welch ANOVA was used. The Kruskal-Wallis H test was applied on not normally distributed data. The subsequent post hoc analyses for significant results were performed with pairwise comparisons using Dunn’s procedure with a Bonferroni correction for multiple comparisons. The chi-square test of homogeneity was used to determine the difference between the binomial proportions of the four groups for dichotomous data. For comparisons over only two groups, we used a Mann–Whitney U test due to not normally distributed data for continuous data and Fisher’s exact test for dichotomous data. Repeated measurements were compared with a Wilcoxon Signed Rank test for continuous and McNemar test for dichotomous data. A two-sided *p*-value of <0.05 was considered as statistically significant. All statistical analyses were calculated using SPSS for Windows, version 22 (SPSS Inc, Chicago, IL, USA) and graphs were drawn with R version 3.6.0.

## 3. Results

### 3.1. Baseline Characteristics

Overall, 80 patients were randomized: 20 (25.0%) to ACE-I, 20 (25.0%) to ARB, 21 (26.3%) to CCB, and 19 (23.8%) to HCT. The mean age was 48 (±14) years, the mean BMI 26.5 (±3.7) kg/m^2^ and 22 (27.5%) were women. The mean 24h systolic BP (sBP) was 141.8 (±9.1) mmHg, diastolic BP (dBP) 87.8 (±7.6) mmHg with no significant differences between groups (Table 1). The baseline characteristics of the normotensive group are included in Table 1.

### 3.2. Angiotensin II (1–8) and Angiotensin (1–7) Equilibrium Concentrations

#### 3.2.1. Comparison between Normotensive Controls and Hypertensive Patients before Treatment Initiation

There were no statistically significant differences regarding Ang II and Ang (1–7) equilibrium concentrations between hypertensives before treatment initiation and normotensive controls (Table 2). Data were missing for one hypertensive at baseline at 8 a.m. and two hypertensives at 12 a.m.

#### 3.2.2. Comparison between the Different Treatment Groups at Baseline and after 4 Weeks of Treatment

##### Angiotensin II (1–8)

There were no statistically significant differences regarding Ang II equilibrium concentrations between the different assigned treatment groups before treatment initiation. However, after four weeks of treatment, there were statistically significant differences regarding the Ang II equilibrium concentrations of patients treated with the four different drugs both before drug intake in the morning and four hours after observed drug intake (Table 3).

**Figure 1:**
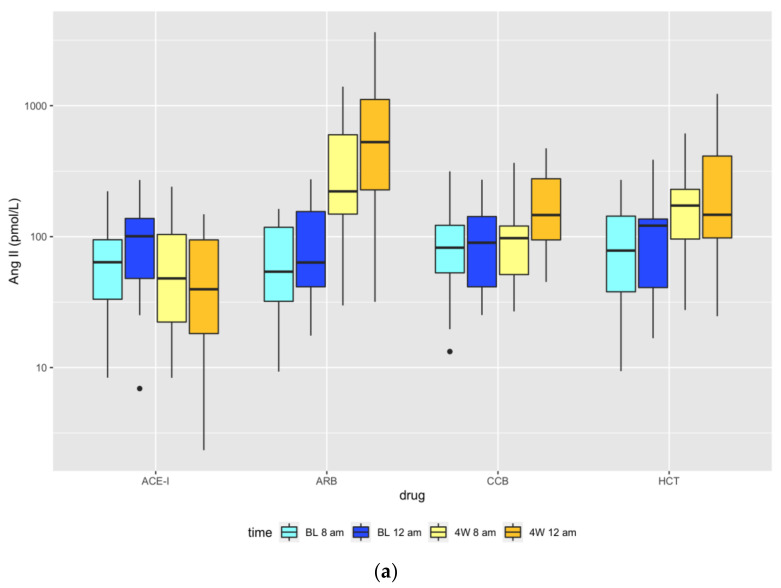

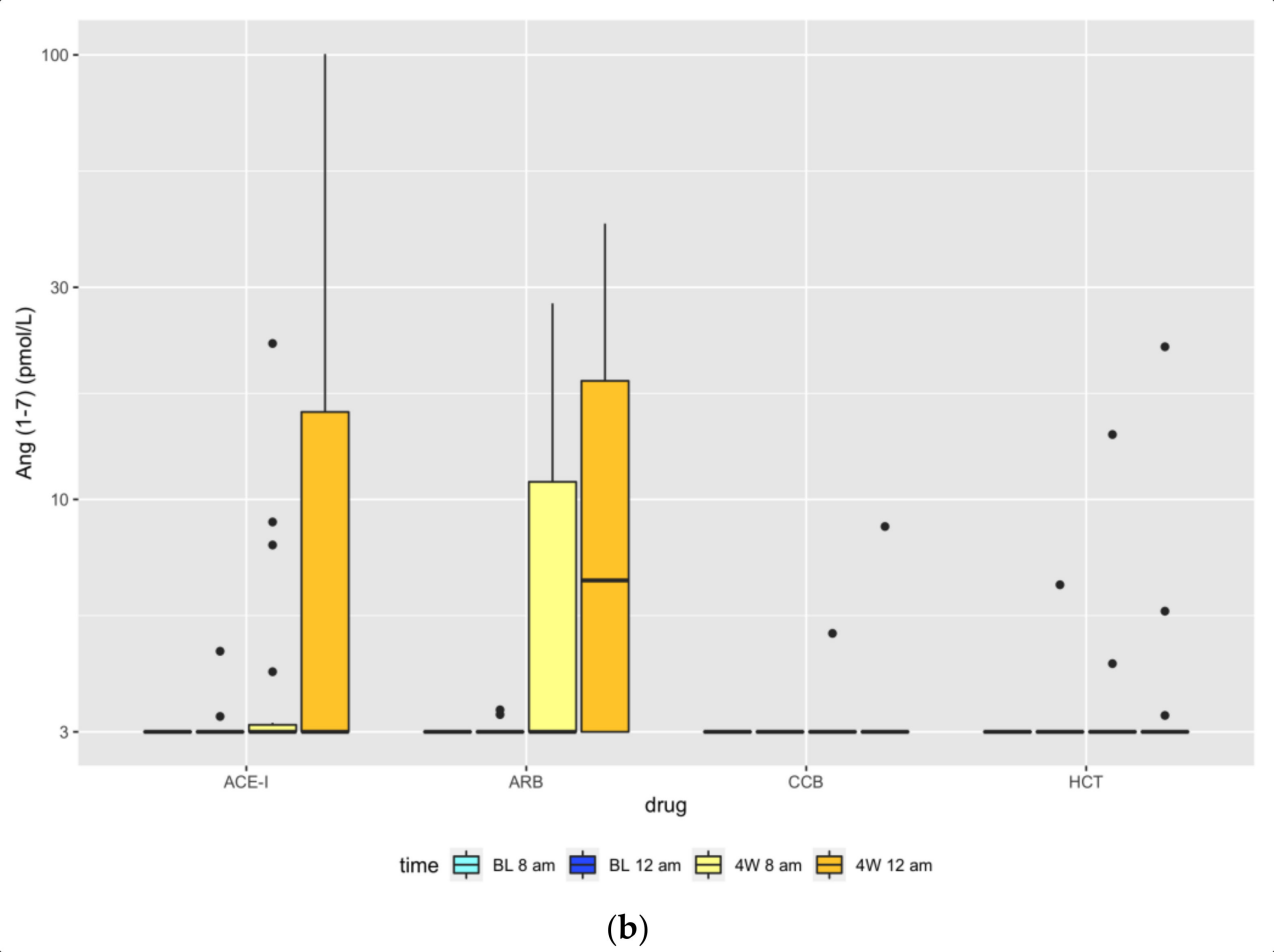
Box plots of Ang II (**a**) and Ang (1–7) (**b**) equilibrium concentrations at baseline (BL) at 8 a.m. (light blue) and 12 a.m. (dark blue), and after four weeks (4W) of treatment at 8 a.m. before drug intake (light yellow) and four hours after observed drug intake (dark yellow). Note: 3 pmol/L is the lower limit of detection for Ang (1–7). Dots correspond to outliers. ACE-I = angiotensin-converting-enzyme inhibitor (perindopril), ARB = angiotensin-receptor blocker (olmesartan), CCB = calcium channel blocker (amlodipine), HCT = hydrochlorothiazide.

Post-hoc pairwise comparisons between the four drugs showed the following significant differences in equilibrium concentrations under treatment at 8 a.m. before drug intake: Ang II was lower with ACE-I than with ARB (*p* < 0.0005), lower with ACE-I than with HCT (*p* = 0.009), and lower with CCB than with ARB (*p* = 0.018). Four hours after observed drug intake, Ang II was significantly lower with ACE-I than with ARB (*p* < 0.0005), CCB (*p* = 0.012), and HCT (*p* < 0.005) (Figure 1a).

##### Angiotensin (1–7)

There were no statistically significant differences regarding Ang (1–7) equilibrium concentrations between the different assigned treatment groups before treatment initiation. However, after four weeks of treatment, there were statistically significant differences regarding the Ang (1–7) equilibrium concentrations of patients treated with the four different drugs both before drug intake in the morning and four hours after observed drug intake (Table 4).

Post-hoc pairwise comparisons between the four drugs showed that under treatment at 8 a.m. before drug intake Ang (1–7) was higher with ARB than CCB (*p* = 0.036). Four hours after observed drug intake, Ang (1–7) equilibrium concentrations were higher with ARB than with HCT (*p* = 0.013), lower with CCB than with ACE-I (*p* = 0.034), and lower with CCB than with ARB (*p* = 0.002) (Figure 1b).

#### 3.2.3. Difference between Baseline and Treatment over Entire Cohort

There were statistically significant differences for the changes of Ang II equilibrium concentrations between baseline and after four weeks of treatment between the four different drugs (Table 5).

Pairwise comparisons showed the following significant differences in 8 a.m. equilibrium concentrations between baseline and under treatment: Ang II equilibrium concentrations were decreased with ACE-I and increased with ARB (*p* < 0.0005), decreased with ACE-I and increased with HCT (*p* = 0.043), more increased with ARB than with CCB (*p* < 0.0005), and more increased with ARB than with HCT (*p* = 0.039). Between baseline at 12 a.m. and under treatment four hours after observed drug intake, we found the following differences: Ang II equilibrium concentrations were decreased with ACE-I and increased with ARB (*p* < 0.0005), decreased with ACE-I and increased CCB (*p* = 0.006), decreased with ACE-I and increased with HCT (*p* = 0.001), more increased with ARB than with CCB (*p* = 0.005), and more increased with ARB than with HCT (*p* = 0.017) four weeks after treatment initiation.

## 4. Discussion

**Figure 2:**
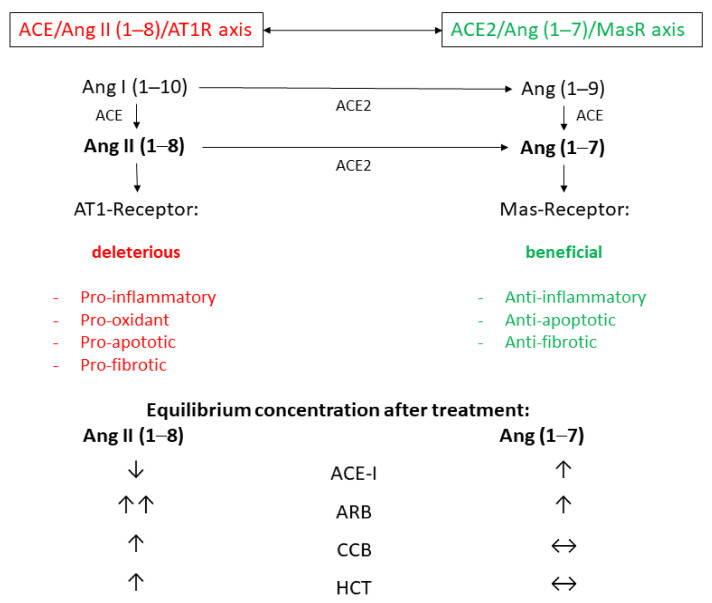
Effect of treatment initiation on Ang II and Ang (1–7) equilibrium concentrations in context of typical effects of each medication. ACE = angiotensin-converting-enzyme, ACE-I = Angiotensin-converting-enzyme inhibitor (perindopril), ARB = angiotensin-receptor blocker (olmesartan), CCB = calcium channel blocker (amlodipine), HCT = hydrochlorothiazide.

In the present study, we identified decisive differences in the equilibrium concentrations of Ang II and Ang (1–7) after four weeks of treating newly diagnosed hypertensive individuals with the four major antihypertensive drug classes ACE-I, ARB, CCB, and HCT, which may have further impact on the RAAS system (Figure 2). Specifically, the ACE-I perindopril led to a decrease in Ang II and an increase in Ang (1–7), whereas the ARB olmesartan led to a massive increase in Ang II and a moderate increase in Ang (1–7). In contrast, the CCB amlodipine and hydrochlorothiazide both increased Ang II, but they showed no measurable effect on Ang (1–7).

Longitudinal changes of Ang II over time showed a significant increase in equilibrium concentrations between baseline and after four weeks of treatment with ARB and HCT both in the morning and at noon, but an increase with CCB and decrease with ACE-I, respectively, was only measureable at noon. These data suggest that these latter may represent short term effects after ingestion of the drug and that peak equilibrium concentrations of ACE-I and CCB are needed to alter Ang II. 

Regarding Ang (1–7), cross sectional data after four weeks of treatment show increased equilibrium concentrations in almost half of the patients under treatment with ARB and ACE-I both in the morning and at noon. This effect was more pronounced at noon. No measureable changes were seen with CCB and HCT, however, due to the quantification limit of Ang (1–7) at 3 pmol/L, changes in patients with very low equilibrium Ang (1–7) may have been missed.

It is known that ACE-I suppress [11,12] and ARB increases Ang II concentrations [13]. Biologically, however, both substances reduce the downstream deleterious effect of Ang II as its AT1R is blocked by ARB. ACE-I interferes with the RAAS at multiple levels. While blocking the conversion of Ang I to Ang II, thereby reducing Ang II levels, the conversion of Ang (1–7) to Ang (1–5) is simultaneously blocked, leading to an accumulation of Ang (1–7). As a consequence, the Ang II/Ang (1–7) ratio, which is frequently used as a marker for ACE2 activity, is reduced by ACE inhibition, mimicking an increase in ACE2 activity. Of note, this purely pharmacologic phenomenon might have significantly contributed to the ongoing discussion of whether ACE inhibitors might increase ACE2 activity. In patients with acute and chronic heart failure, those receiving ACE-I had suppressed Ang II and elevated Ang (1–7) concentrations, but patients treated with ARB had higher Ang II and lower Ang (1–7) concentrations [8]. The effect of HCT on Ang II and Ang (1–7) has been examined in rats, which showed an increase in Ang II concentrations, but no effect on Ang (1–7) [14]. HCT also increases Ang II in humans, which is thought to be due to reduced renal blood flow and thus increased plasma renin concentrations [12,15,16]. However, we are not aware of studies examining the effect on Ang (1–7) in humans. Similar to HCT, an elevation of Ang II under treatment with CCB has been described previously and is thought to be caused by renal blood flow lowering and an increase in plasma renin [17,18,19,20], but to our knowledge, the effect of CCB on Ang (1–7), especially in humans, has not been studied so far.

In the context of the current SARS-CoV-2 pandemic, ACE2 has gained widespread interest, as SARS-CoV-2 utilizes ACE2 for cell entry [21]. This has led to the fear that ACE-I and ARB may facilitate SARS-CoV-2 infection and increase viral load [2]. Therefore, the proposition has been made to switch from RAAS-inhibitory antihypertensives to alternative drug classes [4]. Such a proposition, however, is not ground in scientific evidence and is not supported by the cardiovascular community and major societies [3,22,23,24,25]. Importantly, Ang II may even play a deleterious role in the pathophysiology of severe lung injury and ARDS [26,27,28,29]. Therefore, to assess and weigh the influence of RAAS inhibitors on the cardiovascular and pulmonary system, it is crucial to know both the effects of RAAS inhibitors and of alternative antihypertensive drugs on the RAAS.

ACE2 cleaves the octapeptide Ang II into the heptapeptide Ang (1–7) [5]. Ang II binds to the Ang II type 1 (AT1R) and type 2 receptor (AT2R) [30,31]. The activation of AT1R has been associated with inflammation, vasoconstriction, myocardial and vascular hypertrophy, and fibrosis [32]. Ang (1–7) binds to the Mas receptor (MasR), which antagonizes AT1R mediated effects in most cases [33]. ACE2-depleted mice show increased Ang II concentrations, resulting in severely impaired contractile function, and mild ventricular dilation in the absence of cardiac fibrosis and hypertrophy, without an effect on blood pressure [34]. Upregulation of ACE2 leads to a decrease in Ang II and an increase in Ang (1–7), which exerts protective effects regarding cardiovascular disease and vascular function [5]. Beneath deleterious cardiovascular effects, there is growing evidence that Ang II and Ang (1–7) play an important role in lung injury, too. Ang II induces apoptosis in alveolar cells [27] at least in part via the increase in tumor necrosis factor-alpha (TNF-α) [28] and is involved in fibrogenic processes [35]. The infusion of Ang (1–7) reduced the severity of acute lung injury, inflammation and fibrosis in rodents and was considered a promising therapeutic strategy for the treatment of ARDS-like lung disease [29,35]. In addition, the ARB losartan significantly attenuated TNF-α, interleukin (IL)-6, and IL-1β in mice with ARDS [36] and was also protective in mice with SARS-CoV-associated lung injury [37]. Similarly, the ACE-I captopril mitigated protein leakage, pro-inflammatory cytokine levels and oxidative processes in ventilator-induced lung injury in rats [38,39].

In addition to these pathophysiologic relationships, our study adds that none of the alternative drug classes are indeed neutral on the RAAS in grade I or II hypertensive subjects, even in intermediate dosage and especially regarding Ang II.

Specifically, we observed increased Ang II equilibrium concentrations under treatment with CCB and HCT, which, without the benefit of AT1R blockage (ARB) or increase in Ang (1–7) equilibrium concentrations (ACE-I), may contribute to a pro-inflammatory state and endothelial dysfunction.

### 4.1. Limitations

The RAAS peptide equilibrium concentrations were measured in plasma and we do not have any information on tissue concentrations in general or in the lungs specifically. Additionally, we cannot derive information about the impact on the ACE2 activity or concentration especially in the lungs. It was not possible to calculate Ang II/Ang (1–7) ratios as indirect markers of ACE2 activity since nearly all patients had Ang (1–7) levels beneath the lower limit of detection at baseline. For the ARB, it remains unclear whether the increased Ang (1–7) concentration is caused by a higher ACE2 activity or by the massive Ang II rise and the subsequently increased substrate availability. We used Ang II and Ang (1–7) as markers of the two axes; however, we have no information on the downstream effects of these peptides, which have been reported previously [32,33]. Unfortunately, the number of patients included was too small to calculate differences between subgroups, e.g., male and female patients or different age groups, as both male sex and older age appear to enhance the ACE/AngII/AT1R axis [40]. Further studies are needed on this topic. This study was conducted before the current COVID-19 pandemic, therefore no direct conclusions regarding the effect of antihypertensive medication in the context of COVID-19 outside of pathophysiological considerations can be drawn.

### 4.2. Strengths

The strengths of the study were the thorough evaluation of blood pressure by ambulatory blood pressure measurement as the gold standard for blood pressure evaluation in the intervention group as well as the normotensive group. Additionally, we used a diagnostic liquid chromatography-tandem mass spectrometry/mass spectrometry-based test for the quantification of the RAAS peptides. Unlike ELISA-based methods, LC-MS/MS is a highly specific technology for peptide quantification in complex samples which may overcome the large variations in ELISA and radioimmunoassay methods [41]. To reduce bias caused by possible circadian changes of RAAS peptides, we used specific times for phlebotomy and at the follow up visit observed medication intake.

## 5. Conclusions

The initiation of antihypertensive therapy with intermediate dosages of guideline-based first line drugs causes early and distinct alterations of equilibrium RAAS peptide concentrations. Compared to CCB and HCT, the RAAS peptide equilibrium concentrations under ACE-I and ARB appear more in favor of the Ang (1–7), whereas CCB and HCT are rather stimulatory to Ang II.

## Figures and Tables

**Table 1:** Baseline characteristics in patients randomized for each drug (perindopril (ACE-I), olmesartan (ARB), amlodipine (CCB) or hydrochlorothiazide (HCT)) before treatment initiation and normotensive controls.

Baseline	ACE-I	ARB	CCB	HCT	*p*-Value ^1^	Normotensives
*n* (%)	20 (25.0)	20 (25.0)	21 (26.3)	19 (23.8)		20
Female sex (%)	7 (35.0%)	5 (25.0%)	3 (14.3%)	7 (36.8%)	0.348 ^2^	6 (30.0%)
Age (years) (±SD)	49 (±13)	43 (±16)	48 (±14)	54 (±13)	0.090 ^3^	49 (±13)
BMI (kg/m^2^) (±SD)	26.1 (±3.3)	26.1 (±4.1)	27.0 (±4.0)	26.7 (±3.6)	0.828 ^3^	23.2 (±2.8)
sBP mean (mmHg) (±SD)	139.5 (±7.1)	143.4 (±9.4)	139.3 (±7.2)	145.3 (±11.3)	0.131 ^4^	118.9 (±7.5)
dBP mean (mmHg) (±SD)	87.0 (±6.2)	87.5 (±8.0)	87.3 (±8.7)	89.8 (±7.6)	0.649 ^3^	74.4 (±4.5)
sBP awake (mmHg) (±SD)	145.0 (±7.2)	148.2 (±9.2)	144.4 (±7.9)	150.3 (±10.3)	0.122 ^3^	123.2 (±7.9)
dBP awake (mmHg) (±SD)	91.4 (±6.9)	91.0 (±8.1)	91.8 (±9.5)	93.5 (±6.7)	0.757 ^3^	77.8 (±4.9)
sBP asleep (mmHg) (±SD)	124.9 (±11.3)	130 (±13.0)	125 (±8.9)	132.4 (±15.1)	0.177 ^4^	106.4 (±8.1)
dBP asleep (mmHg) (±SD)	77.1 (±9.5)	77.0 (±10.2)	75.1 (±9.4)	79.2 (±11.7)	0.675 ^3^	63.4 (±5.6)

BMI = body mass index, sBP = systolic blood pressure, dBP = diastolic blood pressure, SD = standard deviation. ^1^ *p*-value comparing the treatment groups: ^2^ chi-Square, ^3^ one-way ANOVA, ^4^ Welch ANOVA.

**Table 2:** Baseline Ang II and Ang (1–7) equilibrium concentrations in the morning (8 a.m.) and at noon (12 a.m.) for hypertensive patients before treatment initiation and normotensive controls, presented as median (interquartile range).

Peptide	Hypertensives *n* = 79	Normotensives *n* = 20	*p*-Value
Ang II 8 a.m. (pmol/L)	68.9 (35.9–116.3)	76.9 (62.3–162.9)	0.141 ^1^
Ang II 12 a.m. (pmol/L)	88.7 (40.0–141.7)	113.5 (68.4–234.3)	0.055 ^1^
Ang (1–7) 8 a.m. (pmol/L)	<3.0	<3.0	1.000 ^1^
Ang (1–7) 8 a.m. (*n* detectable (%))	0 (0.0%)	0 (0.0%)	1.000 ^2^
Ang (1–7) 12 a.m. (pmol/L)	<3.0	<3.0	0.248 ^1^
Ang (1–7) 12 a.m. (*n* detectable (%))	5 (6.4%)	0 (0.0%)	0.580 ^2^

Note: 3 pmol/L is the lower limit of quantification for Ang (1–7). ^1^ *p*-value comparing peptide levels in hypertensives and normotensives calculated using Mann–Whitney U test. ^2^ *p*-value calculated using Fisher’s exact test.

**Table 3:** Ang II equilibrium concentrations at baseline (BL) and during follow up (4W) in the morning (8 a.m.) and at noon (12 a.m.) for each assigned drug, presented as the median (interquartile range (IQR)).

Ang II		ACE-I	ARB	CCB	HCT	*p*-Value
BL 8 a.m. (pmol/L)	median (IQR)	66.5 (28.8–100.2)	54.5 (31.3–122.5)	82.4 (50.5–135.1)	78.3 (36.7–149.3)	0.666 ^2^
4W 8 a.m. (pmol/L)	median (IQR)	48.0 (19.7–111.4)	222.8 (135.0–649.7)	97.4 (46.2–126.1)	172.9 (86.1–234.7)	0.983 ^2^
*p*-value 8 a.m. (BL vs. 4W)		0.313 ^1^	<0.0005 ^1^	0.658 ^1^	0.002 ^1^	
BL 12 a.m. (pmol/L)	median (IQR)	101.2 (40.5–139.3)	63.5 (40.7–164.8)	89.9 (38.9–143.8)	121.3 (38.0–136.9)	<0.0005 ^2^
4W 12 a.m. (pmol/L)	median (IQR)	42.2 (16.1–100.1)	530.1 (201.3–1269.5)	146.2 (88.3–291.5)	146.9 (89.8–451.0)	<0.0005 ^2^
*p*-value 12 a.m. (BL vs. 4W)		0.002 ^1^	<0.0005 ^1^	0.012 ^1^	0.001 ^1^	

ACE-I = perindopril, ARB = olmesartan, CCB = amlodipine, HCT = hydrochlorothiazide. *p*-value calculated using ^1^ Wilcoxon signed rank test, ^2^ Kruskal-Wallis H test.

**Table 4:** Ang (1–7) equilibrium concentrations at baseline (BL) and during follow up (4W) in the morning (8 a.m.) and at noon (12 a.m.) for each assigned drug, presented as the median (interquartile range (IQR)).

Ang (1–7)		ACE-I	ARB	CCB	HCT	*p*-Value
BL 8 a.m. (pmol/L)	median (IQR)	<3.0	<3.0	<3.0	<3.0	1.000 ^3^
	Detectable *n* (%)	0 (0.0)	0 (0.0)	0 (0.0)	0 (0.0)	NA
4W 8 a.m. (pmol/L)	median (IQR)	<3.0 (<3.0–3.1)	<3.0 (<3.0–12.1)	<3.0	<3.0	0.019 ^3^
	Detectable *n* (%)	7 (35.0)	8 (40.0)	1 (5.3)	2 (11.1)	0.021 ^4^
*p*-value 8 a.m. (BL vs. 4W)	For pmol/L	0.018 ^1^	0.012 ^1^	0.317 ^1^	0.180 ^1^	
	For *n*	0.016 ^2^	0.008 ^2^	1.000 ^2^	0.500 ^2^	
BL 12 a.m. (pmol/L)	median (IQR)	<3.0	<3.0	<3.0	<3.0	0.553 ^3^
	Detectable *n* (%)	2 (10.0)	2 (10.0)	0 (0.0)	1 (5.3)	0.747 ^4^
4W 12 a.m. (pmol/L)	median (IQR)	<3.0 (<3.0–16.7)	6.9 (<3.0–28.0)	<3.0	<3.0	<0.0005 ^3^
	Detectable *n* (%)	9 (45.0)	12 (60.0)	1 (5.3)	3 (15.8)	0.001 ^5^
*p*-value 12 a.m. (BL vs. 4W)	For pmol/L	0.008 ^1^	0.002 ^1^	0.317 ^1^	0.109 ^1^	
	For *n*	0.016 ^2^	0.002 ^2^	1.000 ^2^	0.500 ^2^	

Note: 3 pmol/L is the lower limit of quantification for Ang (1–7). ACE-I = perindopril, ARB = olmesartan, CCB = amlodipine, HCT = hydrochlorothiazide. *p*-value calculated using ^1^ Wilcoxon signed rank test, ^2^ McNemar test, ^3^ Kruskal-Wallis H test, ^4^ Fisher’s exact test, ^5^ chi-square test.

**Table 5:** Difference in Ang II equilibrium concentrations between baseline and after four weeks of treatment in the morning (8 a.m.) and at noon (12 a.m.), for each assigned drug, as percentage in comparison to baseline values, presented as median (IQR, *p*-value).

Peptide	ACE-I, Median (IQR)	ARB, Median (IQR)	CCB, Median (IQR)	HCT, Median (IQR)	*p*-Value ^1^
Ang II 8 a.m. (%)	−20.3 (−45.6–33.1)	359.9 (182.9–701.1)	28.7 (−23.3–95.0)	133.8 (9.1–192.1)	<0.0005
Ang II 12 a.m. (%)	−46.1 (−73.5–(−14.8))	607.2 (249.1–1243.0)	59.2 (−5.7–178.3)	52.8 (29.8–243.9)	<0.0005

ACE-I = perindopril, ARB = olmesartan, CCB = amlodipine, HCT = hydrochlorothiazide. ^1^ *p*-value using Kruskal-Wallis H test.

## Data Availability

The data presented in this study are available on request from the corresponding author. The data are not publicly available due to ethical restrictions.

## Supplementary Materials

The following are available online at https://www.mdpi.com/2073-4409/10/3/534/s1, Supplementary Figure S1: Flow chart of study procedures, Data supplement: Inclusion and exclusion criteria.
