# Supplementary material for: Influence of Antihypertensive Treatment on RAAS Peptides in Newly Diagnosed Hypertensive Patients
Source: Cells. 2021 Mar 3;10(3):534. doi: 10.3390/cells10030534 (PMC8001814; doi:10.3390/cells10030534)
Supplement: Supplementary file 1 [file cells-10-00534-s001.zip › Cells supplementary files/Data_supplement1.docx]

*Inclusion criteria:*

- Male or female outpatients with previously untreated non-secondary arterial hypertension, defined as: office-SBP ≥ 140 mmHg and ≤ 180 mmHg and office-DBP ≥ 90 mmHg and ≤110 mmHg.
- Evaluation of 24h blood pressure measurement fulfills criteria of hypertension: mean SBP/DBP ≥ 130/80 on average, ≥ 135/85 during the day, or ≥ 120/70 during the night.
- Age ≥ 18 years
- Body mass index between 18 and 35 kg/m²
- Body weight at least 50 kg
- Ability to understand study procedures and to provide written informed consent
- 12-lead ECG without clinically relevant abnormalities (Exception: signs of left ventricular hypertrophy with increased Sokolow Index).

*Exclusion criteria:*

- Pregnant or lactating women
- Clinical chemistry results indicating secondary arterial hypertension.
- History of or clinically evident cardiovascular disease (other than arterial hypertension), namely myocardial infarction and valvular heart disease or heart failure.
- Patients with ventricular or dual pacemaker
- Uni- or bilateral renal artery stenosis
- Renal dysfunction, defined as estimated creatinine-clearance < 60 ml/min
- Recipient of kidney transplant
- Moderate or severe hepatic impairment
- Clinically relevant lung disease (e.g. uncontrolled bronchial asthma, chronic obstructive pulmonary disease (COPD))
- History of alcohol abuse
- Loss of ≥ 250 ml of blood within 3 months prior to screening.
- Known hypersensitivity to any excipients of the drug formulations
- History or clinical evidence of any disease and / or existence of any surgical or medical condition, which might interfere with the absorption, distribution, metabolism or excretion of the study drugs, or which might increase the risk for toxicity.
- Participation in another clinical trial within past 30 days
- Any circumstances or conditions, which, in the opinion of the investigator, may affect full participation in the study or compliance with the protocol.

Inclusion / exclusion criteria are the same for subjects of the control group except the diagnosis of non-secondary arterial hypertension.
