# Supplementary material for: Influence of Antihypertensive Treatment on RAAS Peptides in Newly Diagnosed Hypertensive Patients
Source: Cells. 2021 Mar 3;10(3):534. doi: 10.3390/cells10030534 (PMC8001814; doi:10.3390/cells10030534)
Supplement: Supplementary file 1 [file cells-10-00534-s001.zip › Cells supplementary files/Supplementary Figure 1.pdf]

Supplementary Figure 1

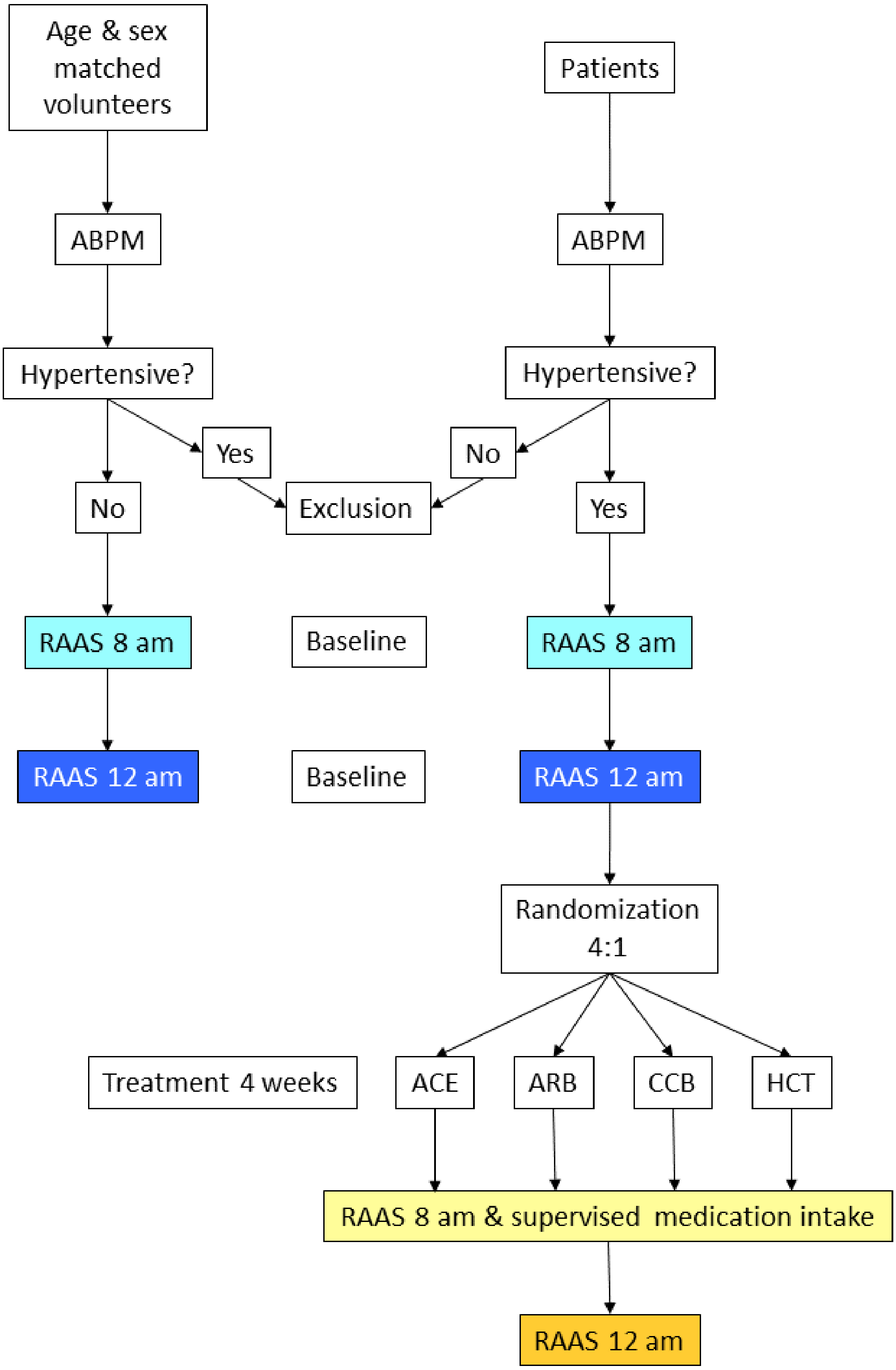

Flow chart of study procedures. ABPM = ambulatory blood pressure monitoring. RAAS = phlebotomy to measure Angiotensin II and Angiotensin (1-7) equilibrium concentrations. ACE-I = Angiotensin-converting-enzyme inhibitor (perindopril), ARB = angiotensin-receptor blocker (olmesartan), CCB = calcium channel blocker (amlodipine), HCT = hydrochlorothiazide.
